# FACILITATORS AND CONSTRAINTS TO SPORT ACTIVITY AMONG ADULTS WITH ARTHRITIS

**DOI:** 10.1093/geroni/igac059.804

**Published:** 2022-12-20

**Authors:** Jill Juris, Julie Son, Jen Wong, Guangzhou Chen, Toni Liechty, Stephanie West, Megan Janke

**Affiliations:** Appalachian State University, Boone, North Carolina, United States; University of Idaho, Moscow, Idaho, United States; The Ohio State University, Columbus, Ohio, United States; University of Illinois at Urbana Champaign, Champaign, Illinois, United States; University of Illinois Urbana-Champaign, Champaign, Illinois, United States; James Madison University, Harrisonburg, Virginia, United States; Berry College, Mount Berry, Georgia, United States

## Abstract

This study examines types of facilitators, constraints, and constraint negotiation strategies and their associations with self-reported physical activity levels for older adults with arthritis. A national sample of U.S. adults (N=288; age range =50-85, M=64.8) who participated in a larger study of sport participation completed an online questionnaire on their involvement in leisure activities. The sample was predominantly White (91.3%), female (65.2%), and unmarried (55.6%). As expected, individuals reporting more constraints engaged in significantly less physical activity (β=-.19, p=.01) while those using greater constraint negotiation strategies reported significantly more activity engagement (β =.18, p=.03). Facilitators were examined (intrapersonal, interpersonal, and structural), but only interpersonal facilitators significantly predicted greater levels of physical activity (β =-.07, p=.03). Adults reporting sport engagement during the past year were also more active (β =.24, p<.001). The discussion will focus on the implications of findings and how barriers to activity in this population can be addressed.

